# Non-Threatening Other-Race Faces Capture Visual Attention: Evidence from a Dot-Probe Task

**DOI:** 10.1371/journal.pone.0046119

**Published:** 2012-10-03

**Authors:** Shahd Al-Janabi, Colin MacLeod, Gillian Rhodes

**Affiliations:** 1 ARC Centre of Excellence in Cognition and its Disorders, Macquarie University, Sydney, Australia; 2 School of Psychology, University of Western Australia, Perth, Australia; Goldsmiths, University of London, United Kingdom

## Abstract

Visual attentional biases towards other-race faces have been attributed to the perceived threat value of such faces. It is possible, however, that they reflect the relative visual novelty of other-race faces. Here we demonstrate an attentional bias to other-race faces in the absence of perceived threat. White participants rated female East Asian faces as no more threatening than female own-race faces. Nevertheless, using a new dot-probe paradigm that can distinguish attentional capture and hold effects, we found that these other-race faces selectively captured visual attention. Importantly, this demonstration challenges previous interpretations of attentional biases to other-race faces as threat responses. Future studies will need to determine whether perceived threat increases attentional biases to other-race faces, beyond the levels seen here.

## Introduction

Organisms are exposed to a large amount of information in the visual environment. It is likely then, that in order to prevent information overload, attention will be selectively captured and/or held by objects that have functional relevance to those organisms [1]. For instance, an organism should be able to quickly detect prey, or notice the physical attributes of a potential mate in order to secure evolutionary goals, whilst disregarding irrelevant stimuli in the environment [2]. Öhman, Flykt and Esteves [3] have demonstrated that this adaptive allocation of attention occurs when people selectively attend to potential sources of harm, such as snakes.

Several researchers have proposed that this prioritisation of attention also extends to social categories related to threat [4], [5], [6], [7]. For example, Trawalter et al. [6] found that images of Black men were afforded preferential attention by White US participants, who typically have negative stereotypes for Black males [8], [9], [10], [11]. In a dot-probe task, White participants were presented with a White and a Black male face for 33 ms, side-by-side on a screen. The participants responded more quickly to a subsequently presented dot probe when it appeared in the locus of the Black face rather than the White face, indicating an attentional bias for the other-race, male faces. This attentional bias has been attributed to the perceived threat valence of Black males [4], [5], [6], [12], [13]. In addition, Trawalter et al. [6] argued for a threat interpretation because no attentional bias was found for Black male faces with averted gaze, which have reduced threat value [12]. Alternatively, however, failure to find a bias with averted-gaze faces may have resulted from reflexive shifts of spatial attention away from those faces (to the gaze direction), thereby reducing task sensitivity to any modulating effects of face race [14].

According to this threat explanation, the attentional bias for other-race faces represents an instance of a more general attentional bias towards threatening stimuli. However, given that these studies used an other-race category that is perceived as threatening, their designs have confounded other-raceness with threat valence. This limitation makes it difficult to determine whether the observed other-race attentional bias is due to the culturally learned message that other-race individuals are potentially threatening, or instead reflects the fact that other-race faces are less familiar, and constitute a more novel visual category than own-race faces. Thus, the first aim of the present research was to test whether an attentional bias to other-race faces depends upon the elevated threat value of such faces, as claimed, by determining whether it extends to non-threatening other-race faces. Attentional bias to non-threatening other-race faces would challenge the claim that those biases represent responses to threat stimuli.

A second aim was to more precisely determine the nature of the attentional bias to other-race faces. Specifically, we investigated whether increased attention to other-race faces reflects a bias in the capture of attention when they appear distal to the currently attended location and/or a bias in the degree to which they selectively hold attention when they appear proximal to the attended location. The dot-probe task used previously to assess attentional responses to other-race faces cannot distinguish these processes because it does not manipulate the initial locus of attention and so cannot control whether faces appear distally from, or proximally to, that location [6]. Therefore, in Trawalter et al's study, slow responses to the dot when it followed an own-race face could reflect either rapid attentional capture to other-race faces, as suggested, or greater attentional hold to those faces. Here we employed a dot-probe variant recently developed to dissociate anxiety-linked differences in the degree to which threat stimuli selectively capture attention, or selectively hold attention [15]. Finally, we assessed the temporal persistence of any attentional biases to other-race faces. It has been claimed that other-race faces may rapidly capture attention, but that this attentional selectivity may not persist for long [16]. We tested this claim by presenting the own-and-other race faces for two exposure durations: 100 ms or 500 ms, following the same approach adopted by previous investigators [17], [18]. The claim would be supported if any observed attentional biases are restricted to the shorter exposure duration.

## Study 1

The purpose of Study 1 was to select an other-race category with low threat value for use in Study 2. White participants were asked to rate White, East Asian and Black male and female faces, together with images of positively and negatively valenced animals. The animal stimuli served as a manipulation check to ensure that ratings were sensitive to threat valence of stimuli.

### Method

**Participants:** Twenty female White students (*M* = 18.1 years, *SD* = 1.7) at the University of Western Australia (UWA) participated for course credit. The study was approved by the ethics committee of UWA and a written informed consent was obtained from the participants in accordance to the Declaration of Helsinki.

**Stimuli:** One-hundred and sixty photographs were used in the rating task: 20 White female faces, 20 White male faces, 20 East Asian female faces, 20 East Asian male faces, 20 Black female faces, 20 Black male faces, 20 positively-valenced animals (e.g. butterflies) and 20 negatively-valenced animals (e.g. sharks). Additionally, eight stimuli, one face of each sex within each race and one from each animal group, were used for the practice trials. The faces were obtained from the UWA FaceLab and Penton Voak's database (2006) at the University of Bristol, and the animal stimuli were supplied by the UWA Cognition and Emotion Lab. Photoshop CS2 was used to scale and align the faces so that the pupils were 80 pixels apart and horizontal. In addition, each stimulus was cropped to remove the background, converted to grey-scale, and equated for contrast and luminance. Each image subtended a visual angle of 4.7°×6.1° from a viewing distance of approximately 50 cm.

**Procedure:** Participants completed 8 practice and 320 test trials. Each stimulus was presented in the centre of the screen, followed by the rating scale. The instructions were to rate how threatening the stimulus looked on a 7-point Likert scale (1 = not very threatening; 7 = very threatening). The scale remained on screen until the participant responded, after which the next trial began. Each image was presented once at each of the two exposure durations that were employed in Study 2 (100 ms and 500 ms). Presentation order was fully randomised.

### Results and Discussion

A paired samples t-test showed that participants rated the negatively-valenced animals (*M* = 6.1, *SD* = 1.8) as more threatening than the positively-valenced animals (*M* = 2.0, *SD* = 1.0), t (1. 19) = 14.21, *p*<.0001, confirming that our rating task was sensitive to stimulus threat valence.

Mean threat ratings for faces were analysed in a 3×2 ANOVA with face race (White, East Asian, Black) and face sex (male, female) as repeated-measures factors. There was a significant main effect of face sex, F (1, 19) = 48.33, *p*<.0001, η^2^p = 0.72, indicating higher threat ratings for male faces (*M* = 4.9, *SD* = 1.5) than female faces (*M* = 3.7, *SD* = 1.3). This result is consistent with previous findings that suggest male faces are perceived as more threatening than female faces [17]. The only other significant effect was an interaction between face race and face sex, F (2, 19) = 6.03, *p*<.01, η^2^p = 0.24.

**Figure 1:**
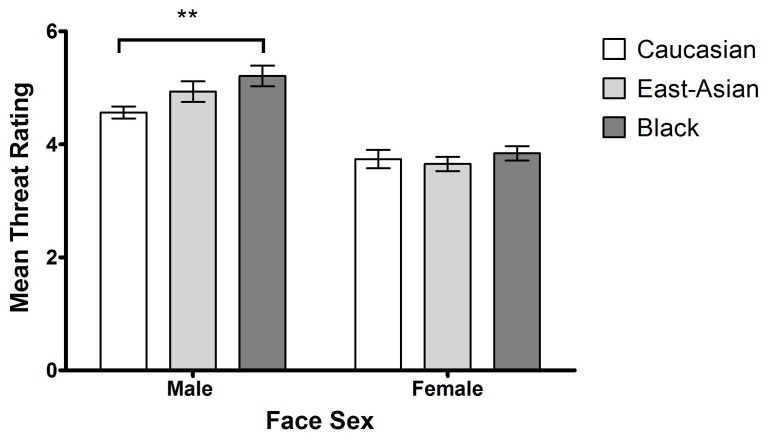
Mean threat ratings for White, East Asian and Black male and female faces (with SE bars). ** p<.01.

The nature of this interaction can be seen from Figure 1. There was a significant simple main effect of face race for the male faces, F (1, 19) = 3.65, *p*<.05, η^2^p = 0.16. Black male faces (*M* = 5.2, *SD* = 1.7) were rated as significantly more threatening than White male faces (*M* = 4.6, *SD* = 1.5), t (1, 19) = 3.05, *p*<.01), replicating the previously established finding that White participants perceive Black male faces as more threatening than male faces of their own race. East Asian male faces (*M* = 4.9, *SD* = 1.7) also were rated marginally more threatening than White male faces, t (1, 19) = 1.79, p = .09.

In contrast, for female faces, there was no significant simple main effect of face race, F (1, 19) = .49, *p* = .62, η^2^p = 0.03, and no significant differences between the threat ratings given to female White, East Asian and Black faces (all ps>.64). Of the two other-race face categories, the ratings given to East Asian female faces were numerically closer to those given to White female faces. Indeed, East Asian female faces (*M* = 3.7, *SD* = 1.3) received precisely the same threat ratings as White female faces (*M = *3.7, *SD* = 1.4). Therefore, we used East Asian female faces as the other-race category in Study 2.

## Study 2

The first aim of Study 2 was to determine whether White participants display an attentional bias to other-race faces that are not perceived as more threatening than own-race faces, namely female East Asian faces. If they do, then it would indicate that attentional bias for other-race faces may not necessarily be responses to threat.

**Figure 2:**
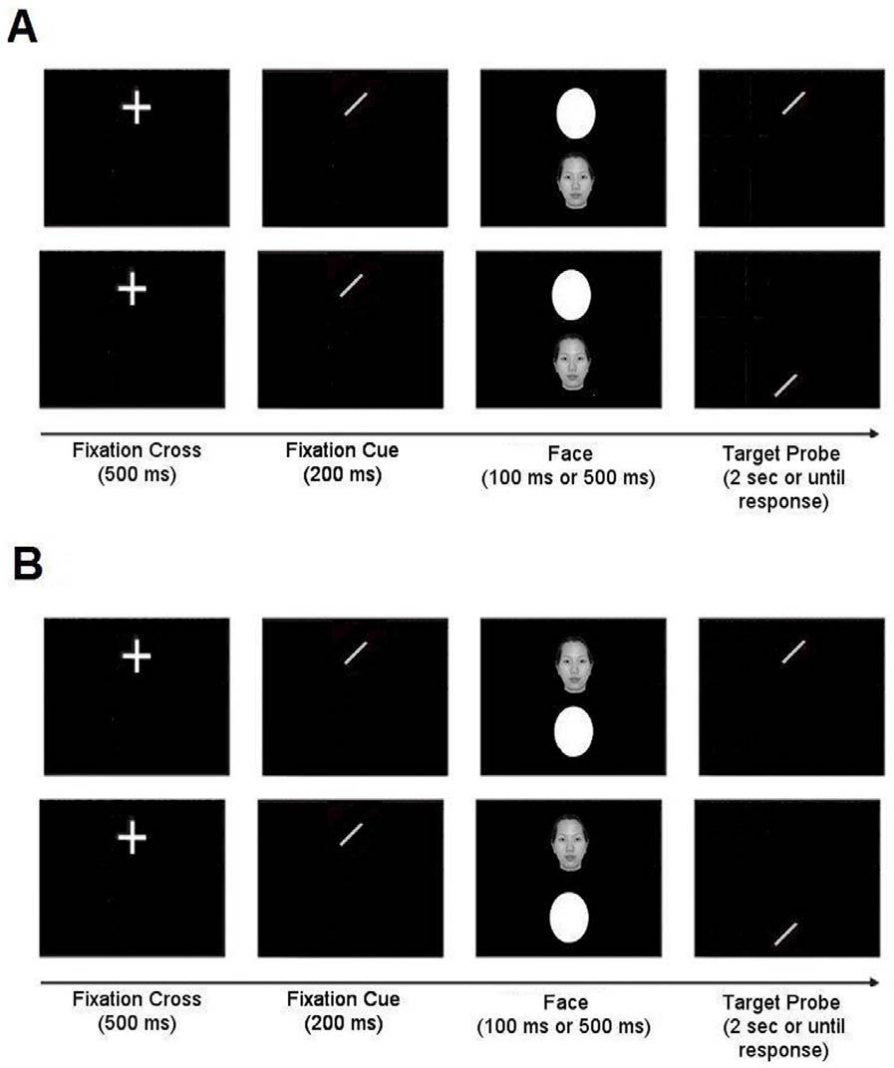
This figure depicts the experimental sequence for Study 1. **A** shows the two sequences of displays for the Face-at-unattended-locus trials: target probe in the attended location (top) and target probe in the unattended location (bottom). **B** shows the two sequences of displays for the Face-at-attended locus: target probe in the attended location (top) and target probe in the unattended location (bottom).

The second aim was to distinguish between enhanced attentional capture by other-race faces, and enhanced attentional holding by other-race faces (Figure 2), using a modified dot-probe task. Specifically, following Grafton et al. [14], we used an early ‘fixation cue’ to control the initial locus of attention on each trial. This method permitted us to assess the capacity of faces to capture attention (through assessing subsequent distribution of visual attention) when they appear distal to this attended location on face-at-unattended-locus trials, and to assess the capacity of faces to hold attention (again through assessing subsequent distribution of visual attention) when they appear at the initially attended location on face-at-attended-locus trials. In both cases, the trial sequence was as follows (see Figure 2): a cross first appeared in an upper screen location indicating where a small line tilted 45° left or right, the fixation cue, would appear. Participants were instructed to attend to the fixation location so that they would be able to register the orientation of the fixation cue for comparison with a subsequently presented line (target probe). The fixation cue was then briefly shown in the fixation location, followed by a face (own-race or other-race), which appeared either in this attended location, or in an unattended location. A neutral white oval simultaneously appeared in the other location. Next, a target probe, a small line small line tilted 45° left or right, was presented either again in the initial fixation (attended) location, or in the opposing (unattended) location. Participants had to indicate whether the orientation of the target probe matched that of the fixation cue.

As in the conventional dot-probe task, relative response latencies for target probes in the attended and unattended locations were used to reveal the distribution of attention across these two locations. Of course, we expected that target probes presented at the unattended location would take longer to process than those presented at the attended location because a shift of attention would be required. Hence, it was on these trials that we expected to find our attentional bias effects. Specifically, on face-at-unattended-locus trials, a greater relative speeding to target probes at the unattended location when these follow other-race than own-race faces, would indicate an attentional capture bias favouring other-race faces. In contrast, on face-at-attended-locus trials, a greater relative slowing to target probes at the unattended location when these follow other-race than own-race faces, would indicate an attentional hold bias favouring other-race faces. We also varied the exposure duration of own-and-other race faces to assess the time course of any attentional biases.

### Method

**Participants**: Twenty female White students (*M* = 18.7 years, *SD* = 1.3) at UWA participated for course credit. The study was approved by the ethics committee of UWA and a written informed consent was obtained from the participants in accordance to the Declaration of Helsinki.

**Stimuli:** The East Asian and White female faces from Study 1 were used. Another twenty faces, ten from each race, were employed in the practice trials. On each trial, a white oval and a single face (both 4.7°×6.1°), were presented on a black background, one above the other, separated by 5 cm from their closest edges. Two versions of each stimulus pair were created, with the positions of the oval and face reversed, so that the face could appear at the top or the bottom. A small (5 mm) red line tilted 45° either to the left or right was used as the fixation cue, and as the target probe. A 1 cm×1 cm white cross was used to cue the position where the fixation cue would appear on each trial. Stimuli were viewed from approximately 50 cm. All stimuli were presented on a black background.

**Procedure:** Participants pressed the space-bar to initiate each of the 80 practice trials and 256 experimental trials. The trial structure (Figure 2) was as follows: a white cross, to which participants were directed to attend, appeared for 500 ms near the top of the screen. The attentional instruction did not explicitly refer to required eye movements (and eye movement measures were not recorded in this study). Rather, participants were free to move their gaze in a naturalistic manner in order to comply with the instructions and perform the task. However, to ensure that attention was initially directed to the specified initial location as required, a fixation cue was then displayed there for 200 ms, immediately after the white cross disappeared. This fixation cue was a small line oriented 45° left or right, with equal probability, on each trial. Immediately following offset of the fixation cue, a face (own-race or other-race) and oval appeared one above the other, with either the face or the oval in the location of the fixation cue, and the other image in the opposite location. After 100 ms or 500 ms, a target probe was presented, again oriented 45° left or right. This stimulus exposure manipulation followed the approach of previous researchers, such as Cooper and Langton [16] - it should be noted that the longer stimulus exposure duration condition also involves a longer onset asynchrony between the face/oval stimuli and the target probe. The target probe appeared with equal probability either in the location where the face or the oval had just been shown. Participants were required to indicate whether the orientation of the target probe matched that of the fixation cue by pressing one of two response buttons. Their orientations matched on 50% of trials. The target probe remained on screen for 2 seconds or until a response was detected.

### Results and Discussion

The dependent variable was mean reaction time for correct responses to the probes (Table 1). Responses more than 2 SD from each participant's mean response time on each condition were removed (1.3% of Face-at-unattended-locus trials and 1.3% of Face-at-attended-locus trials). Accuracy rates were high (*M* = 88.0, *SD* = 8.3). One participant fell more than 2 SD below the mean accuracy and was excluded from further analyses. There was an overall tendency for target probes appearing in the screen locus where the blank oval had just been presented to be responded to more rapidly than those instead appearing where the face had just been presented, t (18) = 4.53, *p*<0.001. We suggest that this pattern may reflect the well-established forward masking effect, whereby the processing of a target stimulus can be slowed when it appears in the same locus as a complex immediately preceding stimulus [19], [20]. In principle, forward masking effects could have been avoided by introducing a delay between the offset of the face/oval stimuli and the onset of the probe. However, to have done so would have potentially compromised our measure of attentional response to the faces, by permitting post face exposure attentional shifts to occur prior to the onset of the target probe designed to assess attentional response to these exposed faces.

**Table 1:** Participants' mean target probe response latencies (ms) for each condition (with standard deviations).

			Face Race			
			White		East Asian	
	Target Probe	Exposure duration	Mean	SD	Mean	SD
Face Locus	Position	(ms)				
Unattended	Attended	100	793	123.1	794	143.9
		500	731	84	759	88.6
	Unattended	100	931	143.2	885	134.8
		500	878	120.9	862	149.9
Attended	Attended	100	837	120.4	800	112.3
		500	794	134.8	780	121.2
	Unattended	100	920	147.5	897	152.3
		500	839	119.3	833	113.2

Of course, this general effect does not tell us whether participants showed differential attention bias to own race versus other race faces, and this issue can be investigated only by examining the significance of interactive effects involving the face race factor. Hence, the response latency data were analysed in a 2×2×2×2 repeated-measures ANOVA, that considered the factors face locus (face-at-unattended-locus, face-at-attended-locus), target probe position (target-at-attended-locus, target-at-unattended-locus), face race (own, other), and exposure duration (100 ms, 500 ms). This analysis revealed a three way interaction between face locus, target probe position and face race, F (1, 18) = 4.65, *p*<.05, η^2^p = 0.21, which was not further modified by exposure duration F (1, 18) = 0.03, *p* = .86, η^2^p = 0.002. Thus, regardless of exposure duration, attentional bias for other-race faces was differentially evident across the conditions designed to assess their ability to capture attention, and to hold attention, respectively. Therefore, in order to understand the nature of this interaction, we undertook separate analyses of the data from face-at-unattended-locus and face-at-attended- locus trials. The outcomes of these analyses are presented below. The same analysis was conducted on the accuracy data, but revealed no significant effects and is not reported here.

### Response Latencies

**Figure 3:**
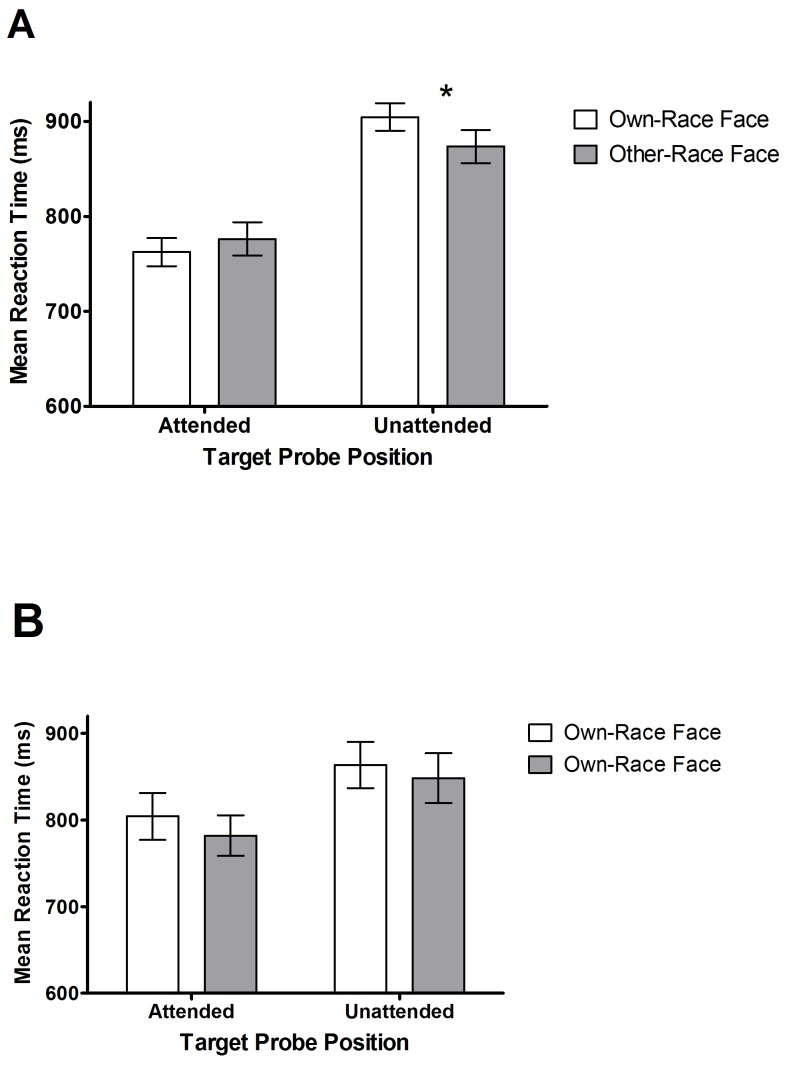
Mean target response latencies for Study 1 are depicted in this figure. **A** shows mean reaction times to the target probe when it follows own - and other - race faces and is presented in the attended and unattended locations (with SE bars) for the Face-at-unattended-locus trials. **B** shows mean reaction times to the target probe when it follows own – and other – race faces and is presented in the attended and unattended locations (with SE bars) for the Face-at-attended-locus trials. * p<0.05.

**Face-at-unattended-locus Trials:** A 2×2×2 ANOVA was conducted on response latencies to the target probes on these trials, which presented faces distal from initial attentional focus. This analysis considered target probe position (target-at-attended-locus vs target-at-unattended-locus), face race (own vs other) and exposure duration (100 ms vs 500 ms) as repeated-measures factors. There was a significant main effect of exposure duration, F (1, 18) = 16.23, *p*<.001, η^2^p = 0.47, due to longer response latencies in the 100 ms (*M* = 851 ms, *SD* = 107) than in the 500 ms (*M* = 808 ms, *SD* = 88) exposure duration condition. There was also a significant main effect of target position, F (1, 18) = 17.02, *p*<.001, η^2^p = .49, with slower responses to target probes that appeared in the unattended location (*M* = 889 ms, *SD* = 127) rather than the attended location (*M* = 769 ms, *SD* = 101) trials. This difference was expected, because in the former case the target probes were distal from initial attentional focus, and thus attention needed to shift to their direction. Of most importance, there was a significant interaction of target probe position and face race, F (1, 18) = 6.00, *p*<.05, η^2^p = 0.25, which was the only other effect to achieve significance (all other Fs<2.81). Figure 3 illustrates this interaction. Not surprisingly, when the target appeared in the attended location there was no significant difference in probe discrimination responses whether the face in the other location was other-race (*M* = 776 ms, *SD* = 108) or own-race (*M* = 762 ms, *SD* = 98), t (1, 18) = 1.24, p = .23. In contrast, when the target appeared in the unattended location, then response latencies were shorter when this face was other-race (*M* = 873 ms, *SD* = 135) than when it was own-race (*M* = 904 ms, *SD* = 125), t (1, 18) = 2.30, *p*<.05, indicating that other-race faces captured selective attention more effectively than own-race faces. There was no significant 3-way interaction with target probe position, face race, and exposure duration, F (1, 18) = 0.01, p = .94, η^2^p = .0001, and hence no evidence that the other-race capture bias attenuates rapidly.

**Face-at-attended-locus Trials:** The same ANOVA was employed to analyse target response latencies from these trials, where the faces were now presented in the location of initial attentional focus. Again there was a significant main effect of exposure duration, F (1, 18) = 26.57, *p*<.0001, η^2^p = .60, with longer target probe response latencies in the 100 ms (*M* = 863 ms, *SD* = 105) than in the 500 ms (*M* = 812 ms, *SD* = 99) condition. Once more, not surprisingly, there was a significant main effect of target probe position, F (1, 18) = 5.6, *p*<.05, η^2^p = .24, reflecting faster response latencies for the target probes presented in the attended (*M* = 803 ms, *SD* = 112) than the unattended location (*M* = 872 ms, *SD* = 125). No other significant effects were present. The main effect of face race fell short of statistical significance, F (1, 18) = 3.95, p = 0.06, η^2^p = .60, and, importantly, there was no significant interaction between target probe position and face race, F (1, 18) = .53, p = .48, η^2^p = .03. Therefore there was no evidence to suggest any attentional holding bias for other-race faces.

### Accuracy Rates

**Face-at-unattended-locus Trials:** We analysed the accuracy rates using the same 2×2×2 ANOVA as above. See Table 2 for mean accuracies. For these accuracy rates, the ANOVA revealed no significant main effect of target probe position, F (1, 18) = 0.27, *p* = 0.61, η^2^p = 0.02, face race, F (1, 18) = 0.36, *p* = 0.56, η^2^p = 0.02, or exposure duration, F (1, 18) = 0.15, *p* = 70, η^2^p = 0.008. Most importantly, the ANOVA revealed no significant interactions, all Fs<0.55.

**Table 2:** Participants' mean accuracy rates (%) for each condition (with standard deviations).

			Face Race			
			White		South-East Asian	
Face Locus	Target Probe	Exposure duration	Mean	SD	Mean	SD
	Position	(ms)				
Unattended	Attended	100	89	9.8	90	10.7
		500	90	10.7	89	10
	Unattended	100	89	7.5	88	10.4
		500	89	8	90	7.9
Attended	Attended	100	89	9.7	92	5.8
		500	91	6	92	7.5
	Unattended	100	88	7.9	88	8.4
		500	87	9	91	8.2

**Face-at-attended-locus Trials:** The same 2×2×2 ANOVA was used to analyse accuracy rates for these trials. For these accuracy rates, the ANOVA revealed only a significant main effect of exposure duration, F (1, 18) = 5.07, *p*<0.05, η^2^p = 0.22, reflecting significantly more accurate target matching responses in the 500 ms condition (*M* = 91, *SD* = 4.26) than the 100 ms condition (*M* = 89, *SD* = 5.16). There was no significant main effect of target probe position, F (1, 18) = 4.25, *p* = 0.054, η^2^p = 0.19, or face race, F (1, 18) = 0.43, *p* = 0.52, η^2^p = 0.02. Again of greatest importance, there were no significant interactions, all Fs<1.76.

The absence of any significant effects of face race on accuracy rates gives grounds for confidence that the significant impact of the face race factor on the observed pattern of probe discrimination response latencies was not due to differential speed accuracy trade-off being adopted as a function of the face race condition.

### Conclusions

Study 1 revealed that White participants do not perceive East Asian and White female faces as differentially threatening. Hence, in Study 2, we measured White participants' selective attentional responses to these East Asian and White female faces, in order to determine whether an attentional bias to other-race faces can occur when those faces are not more threatening than own-race faces. We found that it can. Specifically, the observed pattern of target probe discrimination latencies indicates that East Asian female faces selectively captured White participants' visual attention. This result demonstrates that non-threatening other-race faces can elicit a pattern of privileged attention similar to that found previously using more threatening other-race faces (Black male faces for White participants) [4], [5], [6], [7]. Our findings, therefore, raise the possibility that the attentional biases found in previous studies are unrelated to the processing of threat stimuli. Instead, they may reflect the prioritisation of attention to visual features or stimuli that are relatively novel in the environment (e.g. facial features that deviate from the prototype of faces that one has commonly experienced) [5].

Furthermore, an important and novel contribution of our study was to distinguish between attentional biases in the capture and holding of attention. For the non-threatening other-race faces used here, the pattern of probe discrimination latencies provides evidence for increased attentional capture by other race faces, but not for increased attentional holding by such faces. The capture effect was evident at short as well as long exposure durations, suggesting that the effect occurs rapidly and persists across time. Had it been the case that this attention effect was affected by our exposure duration factor, this could have reflected either the differential stimulus exposure durations, or the differential face/probe stimulus onset asynchronies, associated with this experimental factor. It would be possible, in future research, to manipulate stimulus exposure duration while hold the stimulus onset asynchrony constant (for example by including an inter-stimulus interval of 400 ms following the presentation of the stimulus pair at the short 100 ms duration to equalise the fixation cue-target probe interval for all trials). Of course, this would result in the alternative confound between stimulus exposure duration, and the interstimulus interval separating face offset from probe onset.

Although we have demonstrated that threat is not necessary for other-race faces to capture attention, it is possible that attentional biases to other race faces may be further amplified when participants find these other race faces threatening rather than non-threatening. Study 1 showed that male Black faces were more threatening than female Black faces to White participants. Therefore, if there is a unique contribution of threat over and above visual novelty, we would expect White participants to show larger attentional biases for male than female Black faces. It is also possible that threatening other-race faces, unlike the non-threatening other-race faces used here, might hold attention for longer than own-race faces. This possibility could also be tested in future studies. Furthermore, such future studies could employ implicit measures of the degree to which participants find other race faces threatening, such as the Implicit Association Task [21]. Employing both implicit and explicit measures of perceived threat may clarify whether the attentional capture by (explicitly) non-threatening other race faces seen here could be due to elevated implicit threat value of such faces.

Following Trawalter et al. [6] we tested only White participants and only one other-race category. Like them, we cannot rule out the possibility that some low-level visual property of our other-race (female East Asian) faces, captured attention. However, given that White participants also show attentional biases to another other-race category, Black faces [6], this interpretation seems unlikely. Importantly, our main point is that attentional biases to other-race faces *can* occur in the absence of explicitly perceived threat, and this point stands without further studies. Our findings provide a direct challenge to claims that visual attentional biases to other-race faces necessarily represent biases to threat stimuli, and suggest that the relative novelty of other-race faces may be sufficient to selectively capture attention.
